# Scoping Pharmacy Students’ Learning Outcomes: Where Do We Stand?

**DOI:** 10.3390/pharmacy7010023

**Published:** 2019-02-27

**Authors:** Carla Pires, Afonso Cavaco

**Affiliations:** 1 CBIOS-Universidade Lusófona Research Center for Biosciences and Health Technologies, Campo Grande, 376, 1749-024 Lisboa, Portugal; p5558@ulusofona.pt; 2 iMed.ULisboa, Faculty of Pharmacy, University of Lisbon, Av. Prof Gama Pinto, 1649-003 Lisboa, Portugal

**Keywords:** pharmacy students, pharmacy pharmaceutical undergraduates, learning outcomes, academic performance, education assessment, pharmacy practice, active-learning, learning methodologies

## Abstract

**Background:** The professional abilities of graduate pharmacists have been associated with pharmacy undergraduates’ educational settings and features. This study aimed to perform a scoping review on how students’ learning outcomes are achieved, including learning assessment strategies, focusing on current pharmacy practice education. **Methods:** Relevant keywords, e.g., “pharmacy practice”, “(students or undergraduates)” and “outcomes” were browsed in Public/Publisher MEDLINE, Scientific Electronic Library Online, Directory of Open Access Journals, and other relevant databases for recently published sources (2018 and 2017). Preferred Reporting Items for Systematic Reviews and Meta-Analyses criteria were followed to assure the scoping quality. All types of students’ learning outcomes were addressed for indexed publications in English, Portuguese or Spanish. Reviews, descriptive studies and commentaries were excluded. Study data are presented in tables comprising objectives, methods, number of participants and main research findings. **Results:** Overall, 100 studies were identified and 22 were selected. The selected studies were distributed into seven main topics: real practices (n = 9); active-learning strategies (n = 5); comparisons between different teaching pedagogies (n = 3); pharmacy curriculum (n = 2); and other evaluations (n = 3). **Conclusions:** Studies on pharmacy students’ learning outcomes are limited. Pharmacy undergraduates’ performance was dependent on the learning strategies and extension of syllabus implementation.

## 1. Introduction

In recent decades, there have been significant changes in the pharmaceutical market, such as the arrival of improved technologies, new Internet-based apps and information systems, and innovative medicines and medical devices. It is now possible to diagnose, monitor, advise and provide medical information to patients using mobile electronic-based support systems. Pharmacists are facing new societal and technological challenges, similar to other health professions, and this should be reflected in pharmacy education [1,2]. The International Pharmaceutical Federation (FIP) states that pharmacy and pharmaceutical sciences education “develops a competent, professionally educated pharmaceutical workforce (e.g., pharmacists’ practitioners, pharmaceutical scientists, pre-service students and pharmacy support workforce) for a diversity of settings (e.g., community, hospital, industry and academia)” [3]. 

Currently, academies are required to assure that pharmacy students acquire professional and scientific skills that go beyond the traditional knowledge-centered models [4]. In the old-style learning models, teachers were responsible for transmitting information on a certain topic mainly in lectures, while students were required to listen and study materials, usually after the classes (teacher-centered model). Learner-centered models (LCM) have emerged in recent decades, such as flipped class or team-based learning. These approaches require students to be responsible for learning the course content outside the classroom, while teachers are responsible for guiding students in subsequent practice modules. Flipped classes may be based on cases, problem sets, quiz games, mini-lectures, debates, mind mapping, calculations, review sessions and active-learning inquiries. Team-based learning is a type of flipped classroom where students work in teams or groups to apply knowledge previously acquired outside the classroom [5,6]. In this sense, active training methodologies have been progressively implemented, such as role-plays, video recordings, acting, simulated patients and real interprofessional practices, the last comprising diverse health professionals’ interactions, such as with physicians or nurses [4,7]. The integration of pharmacy students as members of the health care team in real clinical settings, such as hospitals or community pharmacies, with the goal of developing skills and improving patient health, is a common pedagogic approach [8]. On the other hand, some pedagogic initiatives remain limited, such as the incorporation of students’ research into curricula, or requiring students to develop studies and write papers [9]. Overall, these methods have been implemented to achieve a global standard approach addressing the growing number of chronic patients and conditions [10].

Considering that pharmaceutical care has become the most important philosophy of pharmacy practice from the 1990s onwards, in which the gold standard is “the provision of drug therapy for the purpose of achieving definite outcomes that improve a patient’s quality of life”, pharmacy schools should ensure that students are prepared to provide rational drug therapy [11,12]. Indeed, standards in pharmacy education are constantly changing or being updated, namely, with the introduction of diverse competencies in pharmacy degree syllabi, such as professionalism, self-directed learning, leadership and advocacy, interprofessional collaboration, and cultural competencies [4,13]. The study of behavioral sciences is still deficient in some pharmacy practice syllabi, yet student training in skills and attitudes to support personal and professional development have become compulsory in many programs [14]. In addition, the development of undergraduates’ communication skills during pharmacy programs is recommended, since patients’ literacy remains low even in developed countries [14,15]. Appropriate patient–pharmacist communication is essential to prevent misunderstandings and/or medication errors, as well contributing to patients’ treatment adherence, amongst other advantages. The improvement of students’ ability to communicate with patients is classified as a learning outcome already included in many pharmacy curricula [16]. Another aspect is that many schools of pharmacy are trying to find ways not only to improve undergraduates’ professional development, but also to assess and keep track of this process during the pharmacy course, as well as throughout their professional career (i.e., after pharmacy graduation) [10,16,17,18].

Diverse students’ learning outcomes are described in literature, such as students’ acquired knowledge, their self-perception and satisfaction with the curricular programs, or students’ performance in real or simulated pharmaceutical care settings [15,19,20]. Classically, students’ learning outcomes may be classified in three domains, as follows: (i) Knowledge (what students know or understand), (ii) Skills (what students can do or how they apply their knowledge and understanding), and (iii) Competencies (the context in which knowledge and skills can be applied) [21,22]. Moreover, the Center for the Advancement of Pharmacy Education (CAPE) (2013) identified the four domains of students’ learning outcomes as follows: (i) foundational knowledge, (ii) essentials for practicing pharmacy and delivering patient-centered care, (iii) effective approaches to practice and care, and (iv) the ability to develop personally and professionally [23].

The aim of the present study was to perform a scoping review about the evaluation of students’ learning outcomes in current pharmacy practice education, including community pharmacy competences, focusing on recent proposals from Latin-speaking countries, i.e., using Spanish and Portuguese as the learning languages.

## 2. Materials and Methods 

This scoping review was carried out in September 2018 (last online search in 17 September 2018). The *Preferred Reporting Items for Systematic Reviews and Meta-Analyses* (PRISMA) checklist and flow diagram were applied for quality compliance. These criteria are commonly used to report reviews, comprising an evidence-based minimum set of items that should be followed [24]. 

All outputs, such as the types of included/excluded studies, as well as qualitative analyses, were double checked by two independent researchers and discrepancies were solved by consensus. Repeated studies were automatically identified with Endnote Web (EndNote™) (Clarivate Analytics, Philadelphia, PA, USA), an online reference manager [25].

### 2.1. Studies Sources

The searched information sources were Public/Publisher MEDLINE (*PubMed*), Scientific Electronic Library Online (*SciELO*), Directory of Open Access Journals (*DOAJ*), ResearchGate, and the Cochrane Library. *PubMed* is maintained by the National Center for Biotechnology Information as a free resource, with more than 28 million citations [26]. *SciELO* contained 745,182 papers (at the search date), covering a collection of European and Latin-American scientific journals [27]. *DOAJ* is an online directory that provides access to more than 3500 articles of open access and peer-reviewed journals [28]. ResearchGate is a professional network for scientists and researchers, comprising over 15 million members [29], retrieved to also access grey literature. Finally, Cochrane Library is an online collection composed of six databases, including more than 7500 systematic reviews [30].

The present scoping review was focused on finding evidence from Latin-speaking countries but not exclusively, i.e., an international overall contextual perspective was kept. Thus, *PubMed*, ResearchGate and *DOAJ* were selected based on their extensive coverage, but also from a convenience point-of-view. *SciELO* was selected to assure the possible inclusion of studies written only in Spanish and Portuguese. Finally, Cochrane Library was screened to identify previous reviews on the present topic.

### 2.2. Searched Keywords

The searched keywords are presented as a string equation [“pharmacy practice” and (“students or undergraduates”) and “outcomes”] for titles and abstracts. In addition, the Spanish and Portuguese translations of the searched keywords, [“práctica de farmacia” and (“estudiantes and/or universitarios”) and “resultados or desenlaces”] and [“farmácia prática” and (“estudantes ou universitários”) and “resultados or desfechos”], respectively, were screened for the possible identification of papers published in those Latin-based languages. 

Using the broader terms, “(students or undergraduates) and outcomes”, was justified considering that these allow for a wide-ranging search, thus intended to assure the inclusion of the largest possible number of studies in a relatively short period of time. On the other hand, the term “pharmacy practice” was selected to increase the specificity of the present scoping review, since pharmacy practice is one of the most relevant disciplines within the profession and schools of pharmacy, resulting from the fact that community pharmacies are the main source of employment in the pharmaceutical industry around the world [31]. Additionally, the FIP in collaboration with the World Health Organization (WHO) defines a good “pharmacy practice”, as the “practice of pharmacy that responds to the needs of the people who use the pharmacists’ services to provide optimal, evidence-based care” [32]. 

### 2.3. Inclusion and Exclusion Criteria

Studies published in English, Portuguese or Spanish, between 17 September 2016 and 17 September 2018, and addressing any type of pharmacy undergraduate students’ learning outcomes, were included. Exceptionally, other studies, such as Poirier et al.’s [33], were purposively selected, even though pre-health professional students of pharmacy were included (i.e., not pharmacy undergraduates), since undergraduate students from other health professions were also enrolled [33]. A period of 2 years was conveniently defined to ensure the inclusion of the most recent studies and reports. Commentaries, reviews and descriptive studies, i.e., those without a section presenting results from further analysis of a data set were excluded. Due to the scoping nature of this review, some limitations have been identified in the group of selected studies, with study weaknesses globally appraised in the Discussion.

## 3. Results

**Figure 1:**
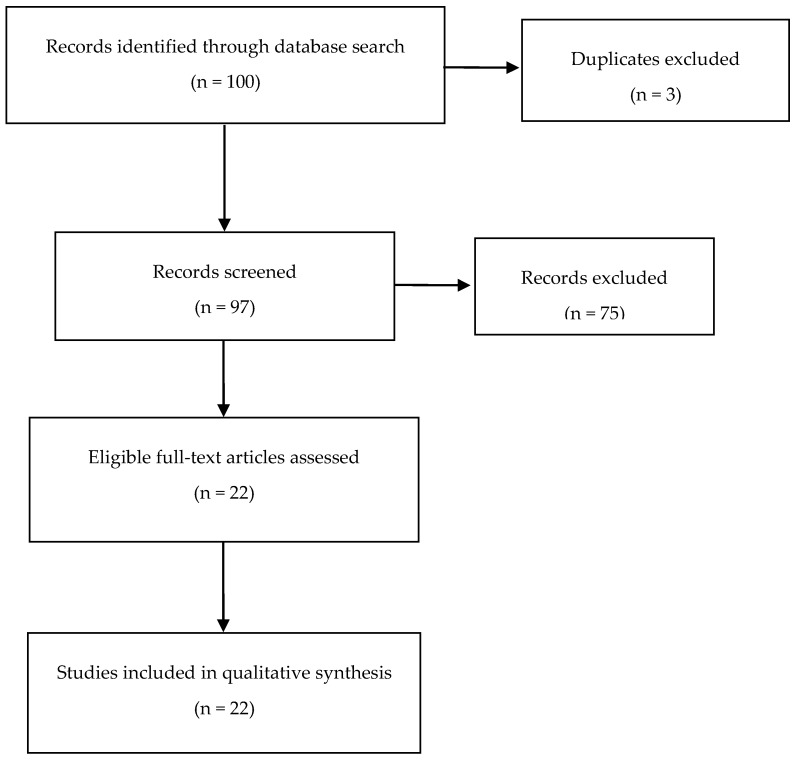
Preferred Reporting Items for Systematic Reviews and Meta-Analyses (PRISMA) (2009) flow diagram identifying included and excluded papers.

Overall, 100 studies were identified: 80 from *PubMed*, 0 from *SciELO*, 0 from *DOAJ*, 20 from ResearchGate, and 0 from *Cochrane Library*. Twenty-two studies were selected as presented in Figure 1. 

### 3.1. Selected and Excluded Studies

The number of selected (n = 22) and excluded (n = 75) studies per source is presented in Figure 1, defined through the PRISMA flow diagram [22]. The reasons for excluding these 75 papers were: reviews studies (n = 14), descriptive studies (n = 6), commentaries (n = 2), other language (n = 1), and other evaluations not covering students’ outcomes (n = 52).

### 3.2. Brief Content Analysis of the Selected Studies

The main study findings, including the year of publication, number of participants/students, methods, results, and conclusions are presented in Table 1 (n = 22 studies).

Seven main research topics were identified in the selected studies, as follows:

1. Learning outcomes in real practice (n = 4);
2. Learning and patients’ outcomes in real practice (n = 5);
3. Learning outcomes using active strategies besides real practice (n = 5);
4. Comparisons between different teaching pedagogies/models (n = 3);
5. Pharmacy curriculum: design of new courses/topics (n = 2);
6. Pharmacy residents as tutors (n = 1);
7. Other evaluations (n = 2).

Table 1 also offers the geographic origin of the selected studies.

## 4. Discussion

Few studies on students’ learning outcomes were identified and selected for this scoping review, which may reflect the limited global research on the present topic. Moreover, no pharmacy higher education institutions, based in countries speaking Spanish or Portuguese, have recently reported their pedagogical innovations concerning pharmacy practice and learning outcomes, although, in Brazil, only 529 pharmacy degrees are officially registered (2016 data) [53].

Most of the selected studies were non-controlled, non-longitudinal, and non-multicentric. None of the selected studies was representative of a certain national population, and the number of enrolled students was very heterogeneous in the included studies. Some studies presented methodological flaws (e.g., no description of sample size) that are critical when interpreting study findings, probably excluding the study from systematic reviews. Additionally, different instruments were applied to collect information on students’ outcomes, such as skills checklists, in-depth interviews, agreement level on Likert scales for different constructs (i.e., tools on self-perceived knowledge, attitudes, communication, interprofessional practice, interdisciplinary education or confidence), or multiple-choice examinations. Contrary to what would be desirable, only some studies have applied pre- and post-surveys, and the number of studies enrolling multiple evaluations was also limited [5,34,35,36,37,38,39,40,41,42,43,44,45,46,47,48,49,50,51,52]. On the one hand, all of these limitations compromise the accuracy and representativeness of the present study findings and conclusions; on the other hand, this clearly shows the need to improve the robustness of learning outcome evaluations in this area.

### 4.1. Real Practice

The studies that evaluated undergraduates’ outcomes in real practice were the most frequently identified, which may reflect the relevance of evaluating students’ performance in actual practice settings [34,35,36,37,38,39,40,41,42], moving from competence acquisition to suitable professional activities (EPAs) [54]. The studies were implemented in health systems, such as medicine services or pharmacies. Around half of these studies did not include the simultaneous evaluation of patients’ outcomes, which may have contributed to an incomplete analysis of students’ learning superiority and performance [34,35,36,37]. 

Undergraduates’ skills, or perception of interprofessional experiences, attitudes and knowledge seemed to improve in real practice [34,35,38,39], although qualitative and quantitative data on students’ performance should be collected during these programs to monitor their progression and adaptation. Particularly, adaptation problems, such as the need for more initial support or delineation of expectations were detected. In addition, students’ intervention seemed to improve patients’ outcomes, such as (i) self-worth, self-care and enjoyment in elder-centered care; (ii) hospital readmissions; or (iii) the health of diabetic patients [38,39,40,41,42]. These findings support the movement towards core EPAs being implemented for new pharmacy graduates in the USA today [54].

### 4.2. Active-Learning Strategies

Students from different course years benefited from participating in active-learning training sessions, such as case scenarios, simulated clinical settings, or role-plays. In some studies, a training/video session or a lecture was provided before the initial laboratory sessions, thus improving students’ prior knowledge. Active-learning strategies successively enhanced students’ self-confidence, identification of drug-related problems, patient management or communication skills [43,45,46,47]. The use of virtual reality and avatars is advancing [55] and has been tested in Portuguese pharmacy undergraduate programs with promising results [56].

### 4.3. Comparisons between Different Pedagogies and Teaching Models

As expected, flipped models have increased students’ performance and knowledge (on pain management), but no advantages seem to be gained by employing team-based learning in comparison with lectures, at least in one study [5,48], although this is not certain [57]. Interestingly, another study has demonstrated that it is possible to increase external dissemination, i.e., the publication of more posters or papers, while developing and implementing optimized practice models and prescribing patterns. This new co-curricular model was composed of diverse courses on research/didactics, research catalogues or practical and experimental projects [49].

### 4.4. Pharmacy Curriculum: Design of New Courses and Topics

Concerning the pharmacy syllabus, only one innovative topic paper and one methodology study were presented within the selected studies. One study focused on the necessity of introducing Medicinal Chemistry into the pharmacy syllabus, as a way of enhancing students’ critical thinking and therapeutic decision-making skills; the other study highlighted the importance of integrating humanistic methodologies in a course for pre-health care students to enhance students’ critical thinking, thus supporting patient care skills [33,50]. There are international guidelines to design quality pharmacy syllabi, including the introduction of useful changes in existing ones, thus inspiring the progress (and reporting) of excellent pharmacy education [58].

### 4.5. Pharmacy Residents as Tutors, and Other Evaluations

The impact of pharmacy residents as instructors seemed to be less educationally relevant, suggesting previous training, namely, on the construction of quality learning objectives [12]. It was also detected limited training in tobacco cessation programs. In addition, possible predictors of students’ performance on the national pharmacy examination were also discussed [51]. Nevertheless, near-peer and peer-led education in different settings has produced good outcomes [59,60]. The last study in Table 1 was the only one applying multivariate regression models, and it concluded that admission criteria and academic performance were the only predictors of learning outcomes [52], suggesting a conservative approach to education attainment.

### 4.6. Limitations of the Scoping Review

As an initial scoping review, the number of included databases and keywords was narrow, which may have limited the present findings. It is not possible to assure the representativeness of all global research published regarding this topic in the considered time interval.

### 4.7. Practical Implications

Although knowing all the limitations when interpreting data from the selected papers, present findings point to benefits emerging from a re-evaluation of curricular programs, at least in the USA. Integrating more real practices and active-learning strategies in pharmacy schools may bring significant benefits, since they may have a direct impact on patients’ health. The provision of educational programs, integrated in real or active-learning practices should be considered; these programs are probably more beneficial if applied from the time of admission, and not towards the end [35,36,37,43,44,48]. The application of flipped and team-based models may be more useful than the traditional expositive models, such as lectures, despite the fact that implementation and outcomes require monitoring [5,48]. If needed, it is possible to increase external dissemination, i.e., students’ scientific outputs, through specific curricular models [49]. Innovative humanistic methodologies may also be systematically considered to increase students’ multicultural background, arguing capacity, and critical thinking [33]. This would be particularly interesting to see in place, knowing that most programs provide clinical training but without necessarily preparing graduates to deal with, e.g., human suffering [61]. Alternative tutors, such as pharmacy residents, should be trained before teaching pharmacy students [12] and could benefit from medical humanities education [51]. The inclusion of a national examination in pharmacy schools to measure the academic curriculum outcomes may be relevant to monitor students’ knowledge [52], especially in other countries besides the USA.

### 4.8. Limitations of the Selected Studies, Future Research and Final Remarks

Additional research on the present topic is recommended, given the social and cultural discrepancies between different educational settings, including anthropological studies and the construction of predictive models. Consequently, the development and validation of standardized tools to evaluate students’ outcomes would be beneficial to a global workforce. The impact of students’ intervention on patients’ outcomes should be evaluated in groups of chronic patients in prevalent diseases, but also those enrolled in lifestyle and behavioral change programs in real practice. It is especially relevant to develop and conduct representative, longitudinal, controlled and multicentric studies, specifically, to design and implement political decisions in pharmacy education.

Considering that some schools of pharmacy are implementing innovative interventions within their curricula, these schools should be encouraged to publish their pedagogies. Additionally, the publication of updated national and international guidelines on teaching/learning strategies is recommended. The findings presented here have limited value to an update of pharmaceutical education guides. The selected studies in this scoping review showed several critical limitations, including a reduced number of participants per study; insufficiently described study designs and/or study protocols; lack of study outcomes monitoring; or not describing the professional experience of the enrolled professors (e.g., number of years). In fact, professors’ performance was not rated or evaluated by the researcher (e.g., other professor). In this sense, governing bodies, such as Center for the Advancement of Pharmacy Education (CAPE) or Accreditation Council for Pharmacy Education (ACPE) may plan or carry out larger studies or reviews to generate updated guidelines and regulations on teaching/learning strategies [14,23]. Besides monitoring and registering undergraduates’ progress, schools of pharmacy should keep track of the professional outcomes and/or knowledge of their graduate pharmacists [17,18].

The teaching/learning strategies identified here seem to have improved students’ learning outcomes. Overall, a positive correlation between a certain teaching strategy and a certain educational outcome seems to exist. However, additional studies are required to check this assumption, knowing, for instance, that perception of interprofessional experiences, attitudes and knowledge of undergraduates seemed to improve in real practice [34,35,38,39]. 

## 5. Conclusions

Studies evaluating undergraduates´ learning outcomes are limited, with only a few specific themes and settings being described. As expected, pharmacy students’ performance and quality assessment were dependent on the teaching strategies and syllabus implementation. Further studies are recommended. 

## Figures and Tables

**Table 1:** Studies included in the scoping review (n = 22).

Authors and Country	Year	Objective	Number of Students	Methods and Results	Main Conclusions
*1.*		*Learning outcomes in real practice*			
Sanders et al. [34] USA	2018	To assess the educational impact of engaging second-year student pharmacists in active, direct patient care experiences in health system practice.	28	Setting: A skill-based four-week introductory pharmacy practice experience in health system practice. Main outcomes: Students were required to complete skills checklists in pre- and post-surveys. Operational and clinical self-efficacy statements were evaluated, e.g., performing proper aseptic technique to compound IV admixtures and gather pertinent patient information from the medical record, respectively. Students also self-identified contributions to patient care. Findings: Significant outcomes were achieved: 81.8% of operational and 100% of clinical self-efficacy statements (*p* < 0.05), and positive perceptions of the program.	Students’ skills were significantly enhanced. It is fundamental to assess data from the experiential education environment to further refine didactic curricula.
Stover et al. [35] USA	2018	To determine students’ knowledge acquisition during an infectious diseases (IDs) advanced pharmacy practice experience (APPE).	40 (5 control)	Setting: IDs consult service at a Level I trauma center and academic medical center. Pre- and post-test evaluations: multiple-choice examination, comprising 50 questions. Main outcomes: Control patients were not integrated in IDs consult. Experimental students were responsible for working with assigned patients daily. These students had access to patient-related pharmacotherapy discussions. Findings: Pre-test scores did not significantly differ between experimental and control students [61.7 (10.9) % versus 62.0 (5.1) %, respectively]. Post-test scores [80.2 (7.9) %] were significantly better than pre-test scores for both experimental and control students.	The IDs APPE improved student performance on a knowledge-based examination. This strategy may be incorporated into pharmacy curricula. Besides experimental education, infectious diseases concepts through coursework and active-learning exercises should be incorporated or strengthened in pharmacy courses.
O’Sullivan et al. [36] USA	2017	To characterize and determine the quality of students’ experience with an attending pharmacist model (APM).	22 Year 1 and 29 Year 2	Setting: Two general medicine services. Longitudinal study of 2 academic years: 2013–2014 (Year 1) and 2014–2015 (Year 2). Main outcomes: Qualitative information collected via in-depth interviews. Quantitative information about student learning and interprofessional interactions also collected. Findings: Strengths and areas needing improvement of APM were identified: some constraints were acknowledged by few students at one site, such as more delineation of expectations, initial support, and initial responsibility.	The APM model is suitable for providing a high-quality learning experience and qualitative results showed precisely the areas needing improvement. APM may be better than the traditional preceptor model for students’ integration in practice. Supervisors should evaluate students’ adaptation and provide educational programs at APM sites.
Tang et al. [37] China	2016	To study curricular effectiveness and impact on students of a community pharmacy experimental course.	61	Setting: 33 pharmacies (and 34 preceptors). Main outcomes: Pre- and post-evaluations of the Community Pharmacy Practice Experience (CPEE) preparatory course. The CPEE comprised the following learning domains: (i) community pharmacy basics; (ii) medications; (iii) dispensing practices; (iv) dietary supplements and health care products; (v) drug information and pharmacy informatics; (vi) pharmacy operation; (vii) pharmacy management; and (viii) off-site elective activities (180 h). A 22-statement, five-point scale questionnaire, was applied before and after the CPEE. Findings: 95.5% of the evaluated ability statements were significantly better self-perceived after the CPPE.	The CPPE was deemed appropriate for teaching community pharmacy practices at the mid-level stage students. It seems that a structured introductory-level CPPE course is valuable for students’ progression. Experimental learning should start from the time of admission.
*2.*		*Learning and patients’ outcomes in real practice*			
Shrader et al. [38] USA	2018	To evaluate the impact on students of interprofessional learning using a practice model, as well as patient outcomes, in ambulatory care.	382 students and 401 patients	Setting: Intervention period: 24-month ambulatory care; 179 students completed the survey instruments. A model was designed to relate students’ practice experience with an interprofessional education curriculum. Main outcomes: Patients’ clinical parameters and students’ professional activities indicators. Findings: Patients’ HbA1c was reduced by 0.5% and screening of depression improved by up to 91%.	Students reported a positive experience and acquired interprofessional collaboration skills. Students’ interventions improved patient clinical outcomes.
Lee et al. [39] USA	2018	To demonstrate the value of training interprofessional students in geriatrics and gerontology within an assisted living facility (elders’ residence).	159 (2014–2015), and 270 (2015–2016)	Setting: Eight sessions on common aging conditions, chronic diseases, and geriatric syndromes (practice), once a month in multiple clinics. Students were placed in interprofessional teams with medicine, pharmacy, nursing and public health students (40% of pharmacy undergraduates); 3–4 students per team, following a minimum of two elders. Main outcomes: After each practice, students and patients were given a five-question Likert-scale survey. Students self-evaluated their communication, knowledge of elder-centered care, understanding of the importance of the clinical topic in delivering elder-centered care, observation of interprofessional collaboration or willingness to participate in future clinics. A similar survey was completed by elders. Findings: Students and patients’ self-evaluations were positively rated.	These practices led to increased perceived knowledge, and improved attitudes and perceptions among students. In addition, self-worth, self-care, and enjoyment increased among the elders. Real-world training in geriatrics and interprofessional team-care of older adults is vital.
Gortney et al. [40] USA	2018	To evaluate the impact of medication histories obtained by students on the identification of medication discrepancies and clinical outcomes.	17	Setting: A total of 215 patients’ medication histories were obtained by 17 students over a 12-month period (students interviewed 148 patients, other professionals interviewed 149 control patients, and both interviewed 67 patients). Main outcomes: Medication histories obtained by students as well as by other health providers were retrospectively compared between students and controls: discharge medication list and 30-day readmissions. Findings: In the period of 30 days after the interviews, there were fewer emergency visits in the student-interviewed group (8 vs. 18; *p* = 0.045).	Medication histories obtained by students improved the information available for identifying inpatients’ drug-related problems, the completeness of the discharge medication list, and reduced the occurrence of emergency department visits within 30 days.
Nagelkerk et al. [41] USA	2018	To improve the health of diabetic patients and practice efficiency within an interprofessional collaborative practice (IPCP).	25 students and 20 staff	Setting: The IPCP involved the completion of educational modules, addressing patient visits, responding to phone calls, team-based case presentations, medication reconciliation activities, and student-led group diabetes education classes. Staff and students agreed on providing consistent patient education during 1 year in a family practice setting. A mixed methods study design was followed. Main outcomes: Results from several tools were obtained, e.g., Interdisciplinary Education Perception Scale (IEPS), the Entry-level Interprofessional Questionnaire, the Collaborative Practice Assessment Tool, and pre/post module knowledge tests. Diabetic indicators were HgbA1c, glucose, lipid, body mass index, blood pressure, and information on annual dental, foot and eye examinations. Qualitative data from focus groups with staff and students were also gathered. Findings: Students and staff significantly improved their knowledge on Team Dynamics and Tips for Behavioral Changes knowledge. Only HgbA1c and glucose levels showed a significant decrease. Interprofessional perceptions were higher at the beginning and did not change throughout the study.	Patients’ outcomes improved in this family practice setting. In addition, students and staff benefited from this program.
Hertig et al. [42] USA	2017	To evaluate students’ performance in a Community Paramedic Program (CPP); assessing drug-related problems identified by students.	11 students and 124 patients	Setting: CPP intended to improve the adaptation of hospitalized patients to home care. Besides pharmacists, 4th-year pharmacy students followed patients in one home visit during the 43-day study period. Main outcomes: Students’ interview data, after a previously assessed role-play. Drug-related problems and other issues with medication at home were identified in home visits. Students provided face-to-face information, and re-evaluated medicines. Findings: From 92 home interviews, 145 drug-related problems were identified, with the most frequent issue being on medicines usage, e.g., continuing hospital medication after discharge. Twenty-two and 25 drug-related problems were identified by students and pharmacists, respectively, in the 15 initial home visits.	Students’ critical thinking and problem-solving skills were consolidated. This program may avoid hospital readmissions due to patients’ inadequate understanding of drug regime changes.
*3.*		*Learning outcomes using active strategies besides real practice*			
Smith et al. [43] USA	2018	To determine the effectiveness of different active-learning exercises in a newly-designed flipped classroom self-care course; the effectiveness in applying the newly acquired knowledge and the improvement of self-confidence to recommend self-care treatments and counsel patients, were also assessed.	208 pre-course and 197 post-course	Setting: Active-learning sessions using case scenarios for non-prescription and dietary supplements intake for 1st-year students. *Main outcomes*: Evaluation data from an anonymous students’ survey, administered pre- and post-course. A final and midterm exam was also applied. Findings: Students self-rated as significantly more confident to develop treatment plans or to counsel patients/family at end of course, although a low performance was registered at the final exam.	Active-learning sessions contributed to increase students’ self-confidence.
Kirwin et al. [44] USA	2017	To design and implement a series of activities focused on developing interprofessional communication skills; to assess the impact of the activities on students’ attitudes and the achievement of educational goals.	130	Setting: Pharmacy practice skills laboratory sessions. Prior to the first pharmacy practice skills laboratory session, a classroom lecture about team communication and short videos about roles/responsibilities/work environments concerning four types of health professionals were administered (registered nurses, physical therapists, nurse practitioners, and dentists). Four subsequent sessions, with role-play, involving a standardized health care professional who asked the students a medication-related question. Main outcomes: Besides students’ performance on the role-play, pre- and post-intervention surveys were administered. Findings: Students’ average scores in all sessions: 90% (SD = 57.4), and survey results showed better student attitudes concerning team-delivered care.	The role-play contributed to improve team communication. The activity was classified as valuable and realistic by students. Students need more exposure to team communication skills, since outcomes from the role-play were poor in some cases.
Bamgbade et al. [45] USA	2017	To evaluate the Willingness to Counsel (WtC) in diabetes, depression and schizophrenia	88	Setting: Third-year pharmacy undergraduates. The study intervention comprised presentations (e.g., on mental illness prevalence, signs and symptoms), videos, discussions and active-learning exercises. Pre- and post-intervention tests were applied. Main outcomes: Data from Link and Phelan’s framework, applied to evaluate the independent variable stigma, namely, comfortability (5 items relating to, or feeling comfortable around, a person with mental illness). Findings: WtC evaluations included medication-related favorable quotes such as “I am likely to screen for medication-related problems in patients with [disease state]”. In the pre-test, diabetes and schizophrenia achieved the highest and lowest WtC scores, respectively. Only the WtC of schizophrenia significantly improved in the post-test. WtC of diabetes was significantly higher than WTCs of depression and schizophrenia in the post-test. Regression results showed that comfortability was a predictor of WtC in both evaluated mental illnesses.	WtC may impact patients’ health; thus, pharmacy schools should support experiential education involving counseling, namely, on mental illnesses.
Hardy and Marshall [46] USA	2017	To discuss course development and results of a survey assessing students’ perceived confidence in performing various skills after course completion.	69 (2010–2011) and 67 (2011–2012)	Setting: Third-year students from 2010 and 2012 were enrolled. All activities were carried out in a fictitious health system, using virtual patients. Twenty-two cases were used in the fall semester and 54 in the spring semester. Main outcomes: Data from a survey applied to evaluate students’ perceived confidence in clinical skills. Findings: Students’ confidence in their clinical skills was improved. Students’ knowledge on therapeutic principles and pharmacotherapeutic recommendations was reinforced.	Students were able to apply knowledge in simulated clinical settings, adding to an increase in their confidence in some of the topics.
Rivkin [47] USA	2016	To describe a student-centered teaching method used to introduce a pharmacist patient care process (PPCP).	85	Setting: A sample of first-year pharmacy students was selected to receive the PPCP. The PPCP was aimed at preparing students for taking medication history, learning to write Subjective Objective Assessment & Plan notes, and patients’ information and drug-related problems assessment. Examples were the “deconstruction” of a patient case or reorganization of patients’ story, as well as identifying a drug-related problem from the medication history. Main outcomes: Data from students’ evaluations comprising multiple-choice examinations, online course evaluations, and the assessment of students’ SOAP notes submissions. Findings: Mean exam question marks ranged from 3.7% to 18.8%, with sampled students’ performance significantly better than the comparative cohort.	Teaching methods were effective. Consistent and systematic delivery of the PPCP may improve students’ skills and confidence, offering a safety environment for introducing patient care into the pharmacy curriculum.
*4.*		*Comparisons between different teaching pedagogies/models*			
Bleske et al. [48] USA	2018	To compare two different teaching methodologies, i.e., team-based learning with lecturing, evaluating the long-term learning outcomes.	30	Setting: Final-year pharmacy students taught in six therapeutic topics, with a sample split into 3 team-based learning groups and 3 traditional lectured groups. Main outcomes: Results from a 47-item questionnaire, six months after course completion. Findings: No statistically significant difference was found between the scores of students from the two different teaching methodologies.	No advantages were gained by employing team-based learning or lectures.
Michalets et al. [49] USA	2018	To compare two co-curricular models in relation to external dissemination rates and preceptor-classified impact on patient care.	65	Setting: The existing co-curricular model was compared to a new model: the longitudinal (12-month) advanced pharmacy practice experience (L-APPE). Among others, the L-APPE model included diverse courses on research didactics and training, and/or the utilization of a research catalogue and a research planning tool. Main outcomes: Patients’ data registered by students enrolled in the new model, as well as projects completion. Posters and peer-reviewed publications were also used as outcome measures. Data from students’ project preceptors gathered through an electronic survey on practice changes. Findings: Posters and peer-reviewed publications had a 350% higher occurrence (RR 4.5, 95% CI 1.9–10.9; *p* < 0.01). L-APPE projects were classified by preceptors 1.5 times more often, leading to a change or confirmation of a practice model or prescribing pattern (83.3% vs. 57.1%; *p* = 0.03).	Besides increasing external dissemination, L-APPE resulted in a more expressive practice model or prescribing pattern benefits.
Lockman et al. [5] USA	2017	To evaluate the impact on learning outcomes of flipping a pain management module.	156 (2015) and 162 (2016)	Setting: First-professional-year (2015 and 2016) involved in a pain management module. The 2015 cohort used the normal model: (instructor-centered), while the 2016 cohort used the flipped model: (learner-centered). The flipped model was based on diverse pre-class activities and in-class active-learning exercises. Pre-class learning activities were ordered as follows: pre-recorded lectures, YouTube-style videos, online interactive modules, case-based guided learning questions, textbooks reading, guidelines reading, review articles reading, and clinical trials reading. Main outcomes: Data collected from both cohorts by two equal assessments at end-of-module, e.g., objective structured clinical examination and multiple-choice exam information. Findings: Learning outcomes significantly improved in the flipped model.	Students’ performance on knowledge- and skill-based assessments was significantly improved by a flipped model on pain management.
*5.*		*Pharmacy curriculum: design of new courses/topics*			
Das et al. [50] USA	2018	To determine students’ perceptions regarding the importance of medicinal chemistry.	112 (2017) and 99 (2016)	Setting: Prospective survey to evaluate the self-perceived impact of incorporating case-based studies in the medicinal chemistry syllabus. Students were asked: (i) how helpful the cases were to enhance interest in medicinal chemistry, and (ii) how positive was the influence of the basic-science knowledge, including their ability to apply the basic principles learned. Main outcomes: Data collected from the evaluation comprising dichotomous replies (yes/no) to the previous questions. Findings: 88% of students from the 2017 class and 92% from the 2016 class responded “yes”.	Demonstrating the connection between foundational medicinal chemistry and its application in pharmacy practice seemed positive. The syllabus can be redesigned, taking into consideration the enhancement of critical thinking and therapeutic decision-making skills through medicinal chemistry principles.
Poirier et al. [33] USA	2017	To design and implement an undergraduate course for pre-health professional students of pharmacy and senior undergraduate students from a variety of majors including pre-medical, pre-dental, nursing, exercise science, and the physical and biological sciences, using a variety of resources from the humanities.	22	Setting: Undergraduate course for pre-health professional students that used literature, films, and podcasts to promote students’ discussion. Focused topics were public health, stigmatization, portrayals of health care providers, patient experiences, health care ethics, aging, and death and dying. A quasi-experimental design was followed. Main outcomes: Data from tasks of reflective writings, a formal written and oral presentation on a selected health-related book, and data from pre- and post-course surveys. Findings: Students’ interpersonal skills improved, as well as their critical thinking, concerning different health care issues.	Humanities could excel in supporting indispensable patient care skill enhancement in students.
*6.*		*Pharmacy residents as tutors*			
Farland et al. [12] USA	2018	To evaluate: (1) students’ performance on subjects taught by 1st and 2nd year postgraduate pharmacy residents (PR) and (2) the quality of the learning objectives and multiple-choice questions developed by the PR.	Students (n = 442): year 1 (n = 170); year 2 (n = 143) and year 3 (n = 129); pharmacy residents: 11 (responsible for content development)	Setting: Pharmacy students enrolled in the Medication Therapy Management course (2010 to 2012). Main outcomes: Data from students’ performance assessments through individual and team readiness assurance tests, and course examinations. The assessment of the quality of the learning objectives and multiple-choice questions written by PR followed pre-defined criteria by authors. Findings: Students performed heterogeneously across the evaluated areas. Twenty (42%) learning objectives and 73 (79%) of the multiple-choice questions met all the quality review criteria.	Impact of resident instructors on student course performance was not educationally significant. Students’ performance varied. Pharmacy residents should be taught to create quality learning objectives that help students to focus on learning the most important course content.
**7.**		**Other evaluations**			
Nduagub et al. [51] USA	2017	To evaluate students’ self-efficacy to provide cessation counseling for cigarette and hookah tobacco.	169	Setting: Training session in the College of Pharmacy on cigarette (82%) and hookah smoking cessation (16%). Main outcomes: Data from an email survey comprising the confidence in counseling and perception of knowledge, based on the Ask–Advise–Assess–Assist–Arrange follow-up (5A’s) model. Findings: Students’ self-confidence in counseling and perception of knowledge was higher for smoking cessation than hookah tobacco.	Training in tobacco cessation is still desirable. Pharmacy students need further training to provide counseling on alternative tobacco products.
Gillette et al. [52] USA	2016	To characterize how independent variables predicted students’ performance on the Pharmacy Curriculum Outcomes Assessment (PCOA) during 1st to 3rd professional years.	Not described	Setting: All students at Marshall University School of Pharmacy participated in surveys, including the PCOA, a national examination used to measure the academic progress of pharmacy students. The PCOA is composed of 4 domains: basic biomedical sciences (16%); pharmaceutical sciences (30%); social, behavioral, and administrative sciences (22%); and clinical sciences (32%). Main outcomes: Data obtained from the Pharmacy College Admissions Test (PCAT), the Health Science Reasoning Test (HSRT), and the PCOA (as a target variable). Findings: PCAT, HSRT, and cumulative pharmacy grade point average were significant predictors of a higher PCOA.	Admission criteria and performance while studying pharmacy were associated with a higher score in PCOA.
